# Índices de Mortalidade por Infarto do Miocárdio Agudo no Brasil – Uma Pequena Luz no Fim do Túnel

**DOI:** 10.36660/abc.20210446

**Published:** 2021-08-09

**Affiliations:** 1 Centro Hospitalar Universitário Lisboa Central Hospital Santa Marta Lisboa Portugal Hospital Santa Marta, Centro Hospitalar Universitário Lisboa Central, Lisboa - Portugal.; 2 Faculdade de Ciências Médicas NOVA Medical School Lisboa Portugal NOVA Medical School - Faculdade de Ciências Médicas, Lisboa – Portugal.

**Keywords:** Infarto do Miocárdio, Mortalidade, Hospitalização, Epidemiologia, Brasil, Estudos de Séries Temporais

Estudos epidemiológicos são o alicerce para o melhor entendimento e planejamento regional da saúde. É igualmente importante implementar programas de avaliação e melhoria da qualidade do serviço de saúde. Para se alcançar este último objetivo, são essenciais os registros prospectivos e análises retrospectivas de práticas clínicas reais. Registros nacionais contínuos podem ajudar a melhoria contínua de qualidade a melhorar no nível do hospital e no nível do país e já demonstraram ser eficazes tanto na Europa quanto na América do Norte^1^. Esses sistemas usam coleta contínua de dados e fornecem relatórios online com foco nos processos de cuidado e nos resultados relacionados a doenças cardiovasculares e intervenções comuns.^1^ Outra abordagem é o uso de dados de bancos de dados de saúde nacionais não dedicados. Entretanto, a maioria dos bancos de dados não foram elaborados para essas análises específicas e as informações são bastante limitadas.

Lucena de Abreu et al.,^2^ apresentam neste periódico um estudo interessante.^2^ Eles analisaram a taxa de mortalidade de pacientes hospitalizados e tratados fora do hospital por infarto do miocárdio agudo (IMA) de 2007 a 2016 em 27 capitais de estados brasileiros, representando aproximadamente 24% da população brasileira. Esse tópico é importante, porque, em uma revisão de periódicos médicos dos últimos dez anos, falta essa informação, e, por esse motivo, a realidade da América do Sul é praticamente desconhecida. As últimas informações disponíveis foram um estudo publicado em 2020 com uma análise retrospectiva de tendências temporais da mortalidade devida ao infarto do miocárdio agudo no Brasil, de 1996 a 2016, que demonstrou uma redução generalizada, especialmente nas capitais, além de revelar desigualdades regionais.^3^

No presente estudo,^2^ os autores analisaram dados do Sistema de Informação sobre Mortalidade do DATASUS. Em uma análise temporal, encontrou-se que as mortes de pacientes internados por IMA, relatadas como índice de mortalidade por 100,000 habitantes, tiveram uma redução muito leve ao longo do tempo. Por outro lado, as mortes de pacientes tratados fora do hospital devido a IMA aumentaram constantemente, mas ainda são mais baixas em comparação às mortes de pacientes hospitalizados. Apesar disso, globalmente, 42% das mortes por IMA foram de pacientes tratados fora do hospital. Os dados demonstraram uma variabilidade muito grande entre as capitais, e houve algumas diferenças em relação às variáveis socioeconômicas entre as mortes de pacientes hospitalizados e tratados fora do hospital. Especialmente, as mortes de paciente tratados fora do hospital foram mais frequentes em homens, octogenários, e solteiros. A mortalidade de pacientes hospitalizados foi inversamente associada ao Índice de Desenvolvimento Humano municipal, o que era um achado esperado, porque um índice mais alto pode estar relacionado a melhores hospitais e qualidade do serviço de saúde. Surpreendentemente, também foi encontrada uma associação direta com os anos de escolaridade esperados, já que os indivíduos com mais anos de escolaridade apresentaram índices de mortalidade mais altos. Era de se esperar que indivíduos com renda financeira mais alta recorressem com mais facilidade a hospitais mais bem equipados, mas esse resultado provavelmente apresenta um viés. Não foi feita nenhuma padronização multivariada, e um impacto negativo possível pode ter resultado de dados das capitais mais importantes, como São Paulo ou Rio de Janeiro, com os índices de mortalidade mais altos, associados a uma população muito grande, disparidades econômicas significativas, e diferenças no acesso aos serviços de saúde, mas também a um número mais alto de anos de escolaridade em comparação a outras capitais. As mortes de pacientes tratados fora do hospital foram inversamente associadas aos anos de escolaridade esperados, como podemos esperar de dificuldades de acesso à saúde, e diretamente associadas às regiões Sul e Sudeste. Essa diferença regional requer uma caracterização completa das regiões para que se possa entender as principais diferenças encontradas entre elas, e isso não é esclarecido no presente manuscrito.^2^

Especialmente relevante, é o achado de que o índice de mortalidade de pacientes hospitalizados e tratados fora do hospital por 100,000 habitantes é extremamente alto nas principais capitais, Rio de Janeiro e São Paulo. Embora se espere que a qualidade do serviço de saúde seja melhor nessas duas capitais, o alto índice populacional, com uma proporção significativa de pessoas de baixo nível socioeconômico, pode estar associado às grandes dificuldades e atrasos no acesso aos serviços de saúde. Outro achado importante é que o índice de mortalidade por IMA em pacientes tratados fora do hospital é mais alto nas capitais do Sul e do Sudeste, e também no Rio de Janeiro.

O estudo^2^ trouxe alguma luz para o entendimento da dinâmica do índice de mortalidade por IMA, especialmente em relação aos pacientes tratados fora do hospital, porque isso ainda não havia sido abordado anteriormente. Entretanto, existem limitações importantes. Como mencionado pelos autores, as causas específicas da morte em pacientes tratados fora do hospital, excluindo morte por trauma ou doenças infecciosas, geralmente não são muito precisas. Faltam dados clínicos, já que a maioria dos casos não é submetida a autópsia, e os atestados de óbito são, na verdade, o resultado de uma conjectura. Em grupos mais idosos, o IMA ou o acidente vascular cerebral são, provavelmente, os diagnósticos mais frequentes em atestados de óbito sem confirmação clínica. É verdade que a maior parte das paradas cardíacas em pacientes tratados fora do hospital é causada por IMA, especialmente quando é possível recuperar informações obre dor precordial prévia. Porém, há outras causas para a dor precordial que são tão letais, ou mais letais ainda, em comparação ao IMA. Se, por um lado, a dissecção aórtica é rara, a embolia pulmonar aguda é bastante frequente, e uma causa muito frequentemente ignorada de dor precordial e parada cardíaca. Além disso, esse estudo revelou a realidade das áreas urbanas, apesar de carecer de representatividade nacional, já que mais de 75% da população brasileira não foi incluída.

Por todos esses motivos, o primeiro estudo abre uma janela sobre o assunto, mas estudos adicionais são essenciais para uma caracterização completa. É importante abordar as desigualdades entre capitais, especialmente em relação ao acesso à saúde, bem como é importante caracterizar totalmente a causa exata das mortes de pacientes tratados fora do hospital. É essencial descobrir até que ponto os atestados de óbito têm vieses. As informações sobre índice de mortalidade devem ser complementadas por dados de registros nacionais, a única maneira possível de se obter informações completas e precisas. Dessa forma, será possível implementar um programa de melhoria de qualidade no país, para abordar desigualdades e otimizar os problemas identificados.
