# Supplementary material for: Índices de Mortalidade por Infarto do Miocárdio Agudo no Brasil – Uma Pequena Luz no Fim do Túnel
Source: Arq Bras Cardiol. 2021 Aug 9;117(2):327–8. [Article in Portuguese] doi: 10.36660/abc.20210446 (PMC8395797; doi:10.36660/abc.20210446)
Supplement: Translation

# Translation: Acute Myocardial Infarction Death Rates in Brazil - A Small Light at the End of the Tunnel

**Affiliations:** 1 Lisboa Portugal Hospital Santa Marta, Centro Hospitalar Universitário Lisboa Central, Lisboa - Portugal.; 2 Lisboa Portugal NOVA Medical School - Faculdade de Ciências Médicas, Lisboa – Portugal.

**Keywords:** Myocardial Infarction, Mortality, Hospitalization, Epidemiology, Brazil, Time Series Studies

Epidemiological studies are the mainstay to better understand and plan regional healthcare. It is also very important to implement programs for quality-of-care evaluation and improvement. To fulfill that last objective, prospective registries and retrospective analysis of real-life clinical practices are essential. Continuous national registries can support continuous quality improvement at the hospital and country level and have proven to be effective in both Europe and North America.^1^ These systems use continuous data collection and provide online reports focusing on the processes of care and outcomes concerning common cardiovascular diseases and interventions.^1^ Another approach is to use data from non-dedicated national healthcare databases. However, most databases were not developed for such specific analysis and information is rather limited.

Lucena de Abreu et al.^2^ present an interesting study in this journal.^2^ They analyzed in-hospital and out-of-hospital death rates by acute myocardial infarction (AMI) from 2007 to 2016 in 27 Brazilian state capitals, representing approximately 24% of the Brazilian population. This topic is important, given that, in a literature review of medical journals over the past ten years, this information is lacking, and, for this reason, the reality in South America is virtually unknown. The last information available was a study published in 2020 with a retrospective review of temporal trends on mortality due to acute myocardial infarction in Brazil from 1996 to 2016, which showed a general decrease, especially in the capital cities, but regional inequalities were also observed.^3^

In the present study,^2^ the authors analyzed data from the Mortality Information System of DATASUS. In a temporal analysis, they found that in-hospital deaths due to AMI, reported as a mortality rate per 100.000 inhabitants, had a very low decrease over time. By contrast, out-of-hospital deaths due to AMI steadily increased, but it is still lower, when compared to in-hospital deaths. Despite this, globally, 42% of AMI deaths were out-of-hospital. Data showed a very large variability between state capitals, and some differences were also found regarding socioeconomic variables between in-hospital and out-of-hospital deaths. Particularly, out-of-hospital deaths were more incident in males, octogenarians, and single individuals. In-hospital death was inversely associated with a higher Municipal Human Development Index, which was an expected finding, because a higher index can be related to better hospitals and quality of care. Surprisingly, they also found a direct association with expected years of schooling, with individuals with longer years of school showing higher death rates. We would expect that those individuals with higher economic income would resort more easily to better equipped hospitals, but this result is probably biased. No multivariate adjustment was made, and a possible negative impact might have resulted from data from major capital cities, like São Paulo or Rio de Janeiro, with the highest death rates, associated with a very large population, important economic disparities, and differences in health care access, but also higher school years compared to other capitals. Out-of-hospital deaths were inversely associated with expected years of schooling, as we might expect from difficulties in healthcare access, and directly associated with South and Southeast regions. This regional difference mandates a full characterization of regions to understand the main differences found between them, and this is not clarified in the present manuscript.^2^

Of particularly relevance, is the finding that both in-hospital and out-of-hospital death rates per 100.000 inhabitants are extremely high in the main capitals, Rio de Janeiro, and São Paulo. Although quality of care is expected to be better in those two capitals, the high population rate, with a significant proportion from a very low socioeconomic background, can be associated with major difficulties and delays in healthcare access. Another important finding is that out-of-hospital death rates by AMI are higher in the South and Southeast capitals, as well as in Rio de Janeiro.

This study^2^ shed some light on the understanding of the dynamics of death rates by AMI, particularly regarding those occurring out-of-hospital, because this has not been previously addressed. However, important limitations are present. As the authors mentioned, the specific cause of death in out-of-hospital deaths, excluding death by trauma or infectious diseases, is usually not very precise. It lacks clinical data, because most cases are not submitted to autopsy and death certificates are in fact the result of an educated guess. In older age groups, AMI or stroke are probably the most frequent diagnosis in death certificates without clinical confirmation. It is true that most out-of-hospital cardiac arrests are caused by AMI, particularly when it is possible to retrieve an information of previous precordial pain. But there are other causes for precordial pain that are as deadly or even more deadly, when compared with AMI. If aortic dissection is rather rare, acute pulmonary embolism is somewhat a frequent and very often overlooked cause of precordial pain and cardiac arrest. This study also showed the reality of urban areas, but it lacks national representativeness, because more that 75% of the Brazilian population was not included.

For all these reasons, this first study opens a window on that subject, but additional studies are essential for a complete characterization. It is important to address inequalities between capitals, particularly in healthcare access, and it is also very important to fully characterize the exact cause of death in out-of-hospital deaths. It is essential to uncover how biased death certificates are. Death rate information should be complemented with data from national registries, the only possible way to obtain complete and accurate information. With this, it will be possible to implement a quality improvement program in the country to address inequalities and to optimize the identified problems.
